# Advancing E-Mental Health in Canada: Report From a Multistakeholder Meeting

**DOI:** 10.2196/19360

**Published:** 2020-04-30

**Authors:** Gillian Strudwick, Danielle Impey, John Torous, Reinhard Michael Krausz, David Wiljer

**Affiliations:** 1 Centre for Addiction and Mental Health Toronto, ON Canada; 2 Mental Health Commission of Canada Ottawa, ON Canada; 3 Division of Digital Psychiatry Beth Israel Deaconess Medical Center Harvard Medical School Boston, MA United States; 4 Department of Psychiatry University of British Columbia Vancouver, BC Canada; 5 University Health Network Toronto, ON Canada

**Keywords:** mental health, psychiatry, medical informatics, digital health, nursing informatics

## Abstract

The need for e-mental health (electronic mental health) services in Canada is significant. The current mental health care delivery models primarily require people to access services in person with a health professional. Given the large number of people requiring mental health care in Canada, this model of care delivery is not sufficient in its current form. E-mental health technologies may offer an important solution to the problem. This topic was discussed in greater depth at the 9th Annual Canadian E-Mental Health Conference held in Toronto, Canada. Themes that emerged from the discussions at the conference include (1) the importance of trust, transparency, human centeredness, and compassion in the development and delivery of digital mental health technologies; (2) an emphasis on equity, diversity, inclusion, and access when implementing e-mental health services; (3) the need to ensure that the mental health workforce is able to engage in a digital way of working; and (4) co-production of e-mental health services among a diverse stakeholder group becoming the standard way of working.

Just before North American travel and face-to-face contact was reduced or eliminated by coronavirus disease (COVID-19), more than 250 people gathered in Toronto, Canada, for the 9th Annual Canadian E-Mental Health Conference [1]. In any given year, 20% of Canadians will have experienced a mental health or addiction related issue [2], and by the age of 40 years, approximately half of Canadians have or will have had a mental illness [3]. With a large number of people requiring access to mental health care and the lack of current face-to-face services in Canada to meet this need, the conference was held to share ideas, leading research and best practices for the use of e-mental health (electronic mental health) technologies to improve care. The conference was founded by the University of British Columbia Department of Psychiatry and coproduced this year by the Mental Health Commission of Canada, University of British Columbia Department of Psychiatry, University Health Network, and the Centre for Addiction and Mental Health. In an effort to ensure the relevancy, timeliness, and potential impact of the topics discussed, the audience was made up of a diverse group of stakeholders such as people with lived experience of mental illness, health providers (including peer support workers, caregivers, and health professionals), researchers, students, administrators, system leaders, vendors/technology developers, and policy makers.

Although there is an abundance of digital technologies used in non–mental health contexts, there are only a limited (but growing) number of examples of meaningful digital technology use among mental health populations in the country [4]. The need to quicken the pace of the adoption of digital technologies within mental health service delivery contexts was discussed. Many strategies were shared. Common themes through all, regardless of technology or tool, were trust, equity, and training. Interestingly, on the heels of the conference, COVID-19 has acted as an unfortunate but much needed accelerator for the scaling and spread of e-mental health technologies [5] such as increasing access to virtual assessments by a physician in Ontario paid for by a temporary billing code of the provincial insurance plan (Ontario Health Insurance Plan) [6]. Watching many of the strategies discussed just weeks before now being adopted locally and worldwide underscores the potential of focusing on trust, equity, and training.

One central theme of numerous discussions was the close attention that needs to be paid to trust, transparency, human centeredness, and compassion in the development and delivery of digital mental health technologies [7]. These concepts are central to the “successful” uptake of these technologies by people with mental illness, especially given the sensitivity of the mental health clinical area. Trust, transparency, human centeredness, and compassion can be understood in several ways. For example, do those using a digital mental health technology trust the information offered digitally? Do they feel the service offered digitally is as good as (or better than) face-to-face service delivery? Do they feel their information is safe and secure enough to share and answer sensitive questions honestly? Do they feel a health professional will do something about their mental health information that is provided digitally? Without being mindful of these important concepts, the risk is that people with mental illness will not use or meaningfully engage with these technologies, and thus, service delivery would have to remain “in person,” which we know is already not meeting the current demand for services.

A second theme that permeated all the workshops and discussions was that of equity, diversity, inclusion and access. These topics must be carefully considered and addressed to ensure that some groups are not disproportionally and/or inadvertency disadvantaged when implementing these technologies. While e-mental health technologies may offer some groups improved quality and access to care, there may be others that are not able to take advantage of this form of care due to accessibility, ability, and language among other reasons. This may have the unintended consequence of creating an even larger equity gap between those who can take advantage of e-mental health technologies and those who cannot. For example, there are still numerous areas of the country with limited internet access and therefore offering e-mental health services to these communities would need to be appropriately supported through addressing significant infrastructure issues. To adequately address important equity, diversity, inclusion and access considerations, it will take new knowledge, specific evidence, and cultural humility to build and adopt smarter approaches to mental health care. Diversity, inclusion, equity, and access are the fundamental starting points for expanding reach and broadening perspectives.

A third theme was that the current mental health workforce is likely not ready at this moment to fully engage in a digital way of working. While it will be important that future mental health professionals like psychiatrists, nurses, and social workers have developed digital health competencies in their entry-to-practice level educational programs, there is a large workforce currently practicing that likely do not possess these competencies. Creative ways to support the development of these competencies is certainly needed, as these mental health professionals are currently practicing within the system and have limited time to engage in formal education. As care is increasingly delivered virtually and uses differing care models than that used today, it is also possible that there are roles that do not exist today (eg, digital specialist or navigator) that may be needed to maximize the potential of digital mental health technologies [8]. Careful consideration must be paid to how the current and future workforce can support a system that is increasingly digital.

In moving the abovementioned agenda forward and improving trust, equity, and training, it will be of great importance to involve all relevant stakeholders (people with lived experience, researchers, health providers, developers, etc) and all relevant communities in coproducing and implementing these e-mental health technologies to ensure success. This is important to ensure the appropriate digital mental health services are developed in the first place, which meet the needs of those who will be using them and are easy to use. Methods for “how to” coproduce and engage diverse stakeholder groups are starting to permeate formal academic literature in the mental health space [9,10]. What is now needed is for these methods to become more commonplace within e-mental health technology implementations. Ideally, co-production, co-design, and meaningful engagement of these relevant stakeholders and communities will become a new standard in which we work.

The group of conference organizers and attendees now have the enormous task ahead of accelerating the lessons learned from the conference during a critical time in recent history. This, of course, needs to be done in a thoughtful way while carefully considering issues of equity, diversity, inclusion, and access. There seems no more urgent a time to ensure that e-mental health technologies are used to deliver necessary care.

For additional information about the conference, review the summary report soon to be published by the Mental Health Commission of Canada.

## Abbreviations

e-mental: electronic mental
COVID-19: coronavirus disease
